# Increased asymmetric dimethylarginine and nitric oxide levels in patients with migraine

**DOI:** 10.1007/s10194-011-0323-7

**Published:** 2011-02-27

**Authors:** Ertugrul Uzar, Osman Evliyaoglu, Gülten Toprak, Abdullah Acar, Yavuz Yucel, Tugba Calisir, Mehmet Ugur Cevik, Nebahat Tasdemir

**Affiliations:** 1 Department of Neurology, Faculty of Medicine, Dicle University School of Medicine, Dicle University, 21280 Diyarbakir, Turkey; 2 Department of Biochemistry, Faculty of Medicine, Dicle University, Diyarbakir, Turkey

**Keywords:** Migraine, Asymmetric dimethylarginine, Nitric oxide, Oxidative stress, Endothelial dysfunction

## Abstract

Asymmetric dimethylarginine (ADMA) has been found as correlated with endothelial dysfunction and oxidative stress. There are few studies regarding ADMA and nitric oxide (NO) levels in patients with migraine and alterations of ADMA and NO levels during migraine attack are not well-known. Therefore, in present study, we aimed to measure NO and ADMA levels in patients with migraine and compare them with the control group to investigate the correlation between migraine, oxidative stress and endothelial dysfunction. The migraine group consisted of 59 patients, including 22 suffering from migraine with aura and 37 suffering from migraine without aura. The control group consisted of 31 healthy volunteers without headache. The patients in migraine group were divided into subgroups based on whether attack period was present or not and whether it was migraine with or without aura. Plasma ADMA levels were measured using an enzyme-linked immunosorbent assay method. Migraine patients had higher concentrations of NO (35.6 ± 7.7, 31.0 ± 6.2 μmol/L, respectively, *p* = 0.005) and ADMA (0.409 ± 0.028, 0.381 ± 0.044 μmol/L, respectively,* p* = 0.001) levels when compared with the healthy controls. During migraine attack, NO and ADMA levels were found to be significantly higher in migraine group as compared to control group (respectively, *p* = 0.015, *p* = 0.014). Similarly, NO and ADMA levels in the patients with migraine in the interictal period were found to be significantly higher as compared to control group (*p* = 0.011, *p* = 0.003). In conclusion, higher ADMA and NO levels of patients with migraine supported that oxidative stress and endothelial dysfunction may have a role in migraine pathogenesis.

## Introduction

Migraine is a common, chronic and disabling neurovascular disorder that characterized by severe headache attacks with photophobia, nausea, vomiting, autonomic symptoms, and in some patients with aura involving neurological symptoms. Although the pathogenesis of migraine is still unclear, different vascular, neurological, and neuroinflammatory mechanisms have been suggested [1, 2]. It has been reported that nitric oxide (NO) plays a role during trigeminovascular inflammation occurring during migraine attacks [2]. Nitric oxide, which is formed by the constitutive isoforms of NO synthase, endothelial nitric oxide synthase (eNOS) and neuronal nitric oxide synthase (nNOS), plays an important roles in the regulation of cerebral blood flow and cell viability and in the protection of nerve cells or fibers against pathogenic factors associated with neurological disorders [3]. Enhanced endothelial NO and superoxide anion release may cause migraine through cerebral blood flow changes. In some studies, oxidative stress due to increased NO has been found especially during attacks in patients with migraine [4, 5]. It is emphasized that neurogenic inflammation, endothelial dysfunction and oxidative stress have a role in migraine pathogenesis [1–5].

Nitric oxide is one of the indicators of oxidative stress [5]. Asymmetric dimethylarginine (ADMA) is nitric oxide synthase inhibitor and ADMA has close association with endothelial dysfunction and oxidative stress [6]. There is limited number of studies investigating ADMA levels in patients with migraine [7, 8]. No difference has been found in ADMA and NO levels between migraine patients during interictal period and healthy controls in a previous study [7]. However, ADMA levels had not been investigated in patients with migraine attack. The association between ADMA and NO in migraine attack is not well-known [7]. For these purposes, in present study, the relationships between migraine, oxidative stress and endothelial dysfunction were investigated by comparing NO and ADMA levels in migraine patients with headache attack and interictal period.

## Patients and methods

A total of 59 (40 women and 19 men) consecutive newly diagnosed migraine patients who did not receive any prophylactic migraine medication were included in this study. The study group consisted of 22 patients, suffering from migraine with aura and 37 migraine patients without aura that followed-up in the outpatient clinic, Department of Neurology, Dicle University Hospital. Migraine was diagnosed based on the criteria of the International Headache Classification II [9]. The patients in migraine group were divided into subgroups as migraine in the attack period (with and without aura) (*n* = 24) and migraine in the interictal period (with and without aura) (*n* = 35). Age range of the patients was 18–57 years (mean ± standard deviation: 33.3 ± 9.4 years). Patients who had hypertension (known hypertension treated with antihypertensive drugs, two or more blood pressure recordings greater than 140/90 mm Hg), coronary artery disease (angiographically proven coronary lesions >50% or documented myocardial infarction, angina pectoris, previous percutaneous coronary intervention or coronary artery bypass grafting), diabetes mellitus (known diabetes treated with diet or drugs or both; or either a fasting serum glucose of more than 126 mg/dl), stroke and renal disease were excluded from the study. The control group included 31 healthy participants without a history of headache**,** vascular diseases, hypertension, diabetes mellitus, and renal and cardiac diseases. The control group comprises 19 women and 12 men. Mean age of the control group was 30.7 ± 8 years (range, 18–50 years). Informed consent was obtained from all participants and the study protocol was approved by the local ethics committee. Blood sampling was performed within 2–5 h from the onset of migraine headache or headache-free period in the patients with migraine patients. Similarly, blood sampling was also performed in healthy individuals. Each collected blood sample was immediately centrifuged at 4,000 rpm +4°C for 10 min and then transferred into an eppendorf tube. Samples were transferred on ice and kept at −80°C deep freeze until the end of the study which was completed in 3 months. The method for plasma nitrite and nitrate levels was based on the Griess reaction [10]. As NO measurement is very difficult in biological specimens, because it is readily oxidized to nitrite (NO^2−^) and subsequently to nitrate (NO^3−^) which serves as index parameters of NO production. Griess method was used as the principle of the NO assay after the protein was removed from the samples by salt precipitation, and the NO^3−^ in the sample was reduced to NO^2−^ by activated cadmium granules, which was equal to the NO concentration of the sample [10]. The plasma ADMA levels were determined using the enzyme-linked immunosorbent assay (Immundiagnostik; Bensheim, Germany) method as previously described [11].

### Statistical analysis

All statistical analyses were performed using the Statistical Package for the Social Sciences (SPSS), version 11.5, for Windows (SPSS, Chicago, IL, USA). Data were expressed as mean ± standard deviation. The normality of the distribution for all variables was assessed by the Kolmogorov–Smirnov test. Student’s *t* test was used for normally distributed variables and Mann–Whitney *U* test was used for non-parametric variables. Relationships between variables were analyzed by Pearson or Spearman correlation analysis according to distribution type of parameters. A *p* < 0.05 was considered to be statistically significant.

## Results

Some demographical and biochemical variables of the study and control groups are shown in Table 1. No differences were found in mean age, body mass index (BMI), blood pressure and levels of fasting blood glucose, cholesterol, triglyceride, LDL and HDL between two groups (*p* > 0.05). The NO and ADMA concentrations of the study subjects are shown in Table 2. Migraine patients had higher concentrations of NO (35.6 ± 7.7 vs. 31.0 ± 6.2 μmol/L, respectively, *p* = 0.005) and ADMA (0.409 ± 0.028, 0.381 ± 0.044 μmol/L, respectively, *p* = 0.001) when compared with healthy controls. NO and ADMA concentrations of the migraine subgroups are shown in Table 3. No statistically significant difference was found between NO (35.3 ± 6.4, 35.8 ± 8.5 μmol/L, respectively, *p* > 0.05) and ADMA (0.407 ± 0.025, 0.410 ± 0.031 μmol/L, respectively, *p* > 0.05) levels during attack and interictal period in migraine group. No statistically significant difference was determined between ADMA (0.410 ± 0.033 vs. 0.406 ± 0.017 μmol/L, *p* > 0.05) and NO (35.9 ± 8.4 vs. 35.0 ± 6.5 μmol/L, *p* > 0.05) levels of the patients with aura as compared to the patients without aura in migraine group. No statistically significant correlation was found between age, gender, attack frequency, disease duration and NO and ADMA levels in migraine group (for each parameter, *p* > 0.05).

**Table 1:** Demographical and biochemical variables of the study and control groups

	Migraine patients (*n* = 59)	Controls (*n* = 31)	*p* values
Age (years)	33.1 ± 9.9	30.7 ± 8.1	N.S.
Sex (M/F)	19/40	12/19	N.S.
HDL-C (mg/dl)	48.4 ± 13.6	50.1 ± 8.8	N.S.
LDL-C (mg/dl)	102.1 ± 23.8	95.7 ± 34.1	N.S.
Cholesterol (mg/dl)	174.6 ± 32.4	176.8 ± 31.2	N.S.
Fasting glucose (mg/dl)	92.7 ± 6.1	91.2 ± 4.9	N.S.
Systolic blood pressure (mmHg)	108.3 ± 12.3	106.1 ± 10.8	N.S.
BMI (kg/m^2^)	26.2 ± 2.6	27.1 ± 3.2	N.S.

*N.S.* statistically not significant (*p* > 0.05), *BMI* body mass index

**Table 2:** Comparison of nitric oxide (NO) and asymmetric dimethylarginine (ADMA) levels of control and migraine groups

	Migraine patients (*n* = 59)	Controls (*n* = 31)	*p* values
NO (μmol/L)	35.6 ± 7.7	31.0 ± 6.2	0.005
ADMA (μmol/L)	0.409 ± 0.028	0.381 ± 0.044	0.001

**Table 3:** Comparison of nitric oxide (NO) and asymmetric dimethylarginine (ADMA) levels of migraine subgroups

	Migraine during attack (*n* = 25)	Interictal period (*n* = 24)	*p*
NO (μmol/L)	35.3 ± 6.4	35.8 ± 8.5	N.S.
ADMA (μmol/L)	0.407 ± 0.025	0.410 ± 0.031	N.S.
	Migraine with aura (*n* = 22)	Without aura (*n* = 37)	P
NO (μmol/L)	35.0 ± 6.5	35.9 ± 8.4	N.S.
ADMA (μmol/L)	0.406 ± 0.017	0.410 ± 0.033	N.S.

*N.S.* statistically not significant (*p* > 0.05)

## Discussion

Our study showed that plasma concentrations of ADMA and NO were elevated in migraine patients as compared to control subjects. The migraine during the headache attacks does not lead any significant difference in ADMA and NO levels as compared to the migraine during interictal period.

Nitric oxide, a labile molecule with a half life of only a few seconds is synthesized generally in the endothelium. It is rapidly oxidized by tissue oxygen to the stable end products, nitrate and nitrite. Thereby, to best index of overall NO production in the circulation, is the total concentrations of both nitrate and nitrite [5, 6]. NO is one of the substances that takes a part in the pathophysiology of migraine. NO is involved in the regulation of the cerebral vessels tone. NO may function as a signaling molecule in controlling neuronal activity and is important in controlling sensory inputs during migraine attack and interacting with reactive oxygen substances (ROS), which may induce headache through changes of cerebral blood flow [12]. The most important effect of NO is the activation of the soluble guanylate cyclase. This enzyme causes the synthesis of cyclic guanosine monophosphate (cGMP) and NO-cGMP pathway in smooth vascular muscles causes vascular dilation and relaxation [13, 14]. It is suggested that NO could be an important mediator in the initiation or the propagation of a neurogenic cranial vessel inflammatory response that might eventually result in a migraine attack [14]. It has been found that the pathways consisting of NO synthesis are activated in the experimental headache models such as nitroglycerin-induced headache [15]. The increase of NO may be caused by interactions with free ROS. The primary product of the interaction between NO and superoxide anion is peroxynitrite. Peroxynitrite is an aggressive and potent cellular oxidant [16]. So, increased NO with migraine patients may be associated with oxidative stress. In addition, occurrence of peroxynitrite following NO increase affects endothelia function [17]. Marked NO increase in migraine group in our study strengthened the correlation between oxidative stress and endothelia dysfunction in pathophysiology of migraine. Likewise, Yılmaz et al. [5] suggested that NO may serve as useful markers to show the increased oxidative stress in migraine patients. Taffi et al. [18] found significant increased peroxynitrite as a potent oxidant in platelets of patients with migraine without aura during headache-free periods. It has been reported that the migraine headache and the changes in cerebral blood flow during migraine attacks may be related to the production of NO [16, 19]. Also, Shimomura et al. [20] found that high concentrations of platelet nitrate/nitrite ratio during migraine attacks. But, Shukla et al. [21] found no changes in polymorphonuclear leukocyte, platelet and plasma nitrite levels in patients with migraine. Additionally, it is suggested that intranasal hydroxocobalamin, a NO scavenger, may have a prophylactic effect in migraine [22]. Highly increased NO levels of our migraine patients during attack and interictal period as compared to the controls are in concordance with the results of the most studies [16–20].

In recent studies, a relationship between migraine, stroke and cardiovascular diseases have been reported [23, 24]. Endothelial dysfunction is induced by numerous cardiovascular risk factors and represents an early event in the development of atherosclerosis [25]. ADMA are elevated in several disease states, including those characterized by endothelial dysfunction such as stroke, cardiovascular diseases, hypertension, pre-eclampsia, atherosclerosis, hypercholesterolemia, diabetes mellitus, and chronic kidney disease [25, 26]. It is suggested that endothelial dysfunction has a role in the pathogenesis of correlation between migraine and stroke [24]. Therefore, in our study, ADMA level, a marker of endothelial dysfunction, was measured in migraine cases. To the contrary of two previously conducted studies, ADMA levels were found to be significantly higher in migraine cases compared to the control group in our study [7, 8]. This finding supports the roles of oxidative stress and endothelial dysfunction in migraine pathogenesis. Our study is the first study determining ADMA elevations in migraine. Possible reasons for increase in levels of both NO and ADMA in the patients with migraine in our study may be as follows: (1) decrease in activity of dimethylarginine dimethylaminohydrolase (DDAH) enzyme, metabolizing ADMA and/or (2) excess amounts of ADMA could have been produced to compensate the abnormally increased NO in the body. It has been demonstrated that DDAH activity is regulated by NO [8, 27]. This implies that under certain conditions when NO generation increases, DDAH activity may be diminished. This would lead to accumulation of ADMA and inhibition of NO synthase [26]. Therefore, it is conceivable that an increase in ADMA in migraine might represent a compensatory mechanism to decrease NO production and consequently, to counterbalance excessive vasodilatation. It is suggested that endothelial injury, impaired endothelial vasoreactivity and increased carotid artery intima media thickness occur with migraine and these are associated with vascular risk factors [28]. In some studies, particularly migraine with aura has been found to be associated with endothelial dysfunction. It has been reported that there is evidence of increased endothelial activation in premenopausal women with migraine, particularly in those with migraine with aura, [29]. In a previous study, an eNOS (Glu298Asp) gene polymorphism decreasing eNOS activity has been determined more frequently in migraine patients with aura [30]. In our study, ADMA levels of migraine patients both with and without aura were found to be markedly higher than those of the healthy controls. When ADMA levels of migraine patients with and without aura were compared, slightly higher but not significantly different ADMA levels were found in patients without aura. These findings indicate the possible endothelial dysfunction in migraine patients both with and without aura. Findings of increased ADMA and NO in migraine may lead to new therapeutic strategies including probable antioxidant use for management of migraine In the future, development and enhancements of existing drugs may be accompanied by increased efforts to develop truly new migraine drugs based on knowledge of the pathophysiology [31, 32].

In conclusion, increased ADMA and NO levels of migraine patients support the roles of oxidative stress and endothelial dysfunction in migraine pathogenesis. Further clinical and biochemical studies are needed to investigate the associations between migraine, stroke and ADMA and to introduce new therapeutic modalities for migraine treatment.
